# Identification and monitoring of coal dust pollution in Wucaiwan mining area, Xinjiang (China) using Landsat derived enhanced coal dust index

**DOI:** 10.1371/journal.pone.0266517

**Published:** 2022-04-08

**Authors:** Nan Xia, Wenyue Hai, Gimei Song, Mengying Tang

**Affiliations:** 1 College of Geographical Science, Xinjiang University, Urumqi, Xinjiang, The People’s Republic of China; 2 Xinjiang Key Laboratory of Oasis Ecology, Xinjiang University, Urumqi, Xinjiang, The People’s Republic of China; 3 Key Laboratory of Smart City and Environment Modelling of Higher Education Institute, Xinjiang University, Urumqi, Xinjiang, The People’s Republic of China; Duy Tan University, VIET NAM

## Abstract

Coal dust is the main pollutant in coal mining areas. Such pollutants easily diffuse and are difficult to monitor, which increases the cost of environmental pollution control. Remote sensing technology can be used to dynamically monitor mining areas at a low cost, and thus, this is a common means of mining area management. According to the spectral characteristics of various ground objects in remote sensing images, a variety of remote sensing indexes can be constructed to extract the required information. In this study, the Wucaiwan open-pit coal mine was selected as the study area, and the Enhanced Coal Dust Index (ECDI) was established to extract the coal dust pollution information for the mining area. A new mining area pollution monitoring method was developed, which can provide technical support for environmental treatment and mining planning in Zhundong. The results of this study revealed the following: (1) Compared with the normalized difference coal index, the ECDI can expand the difference between the spectral information about the coal dust and the surrounding features, so it has a significant recognition ability for coal dust information. (2) From 2010 to 2021, the coal dust pollution in the study area initially increased and then decreased. With the continued exploitation of the coal mines in the study area, the coal dust pollution area increased from 14.77 km^2^ in 2010 to 69.49 km^2^ in 2014. After 2014, the local government issued various environmental pollution control policies, which had remarkable results. The coal dust pollution area decreased to 36.85 km^2^ and 17.85 km^2^ in 2018 and 2021, respectively. (3) There was a great deal of pollution around mines and roads, around which the pollution was more serious. Various factors, such as wind, coal type, and the mining, processing, and transportation modes, affect the distribution of the coal dust pollution.

## 1 Introduction

Coal dust refers to all of the forms of fine mineral particles produced during coal mining, processing, production, and transportation. Coal dust pollution is hazardous to human health. Various respiratory diseases, such as pneumoconiosis, are developed when miners inhale a large amount of coal dust during coal mining [1–4]. An explosion or fire is likely to occur when the concentration of coal dust particles reaches a critical value in the air [5–7]. Coal dust also has a large impact on the environment. For example, if coal dust falls on plant leaves, it will block the pores and inhibit photosynthesis and respiration, which will endanger the health of vegetation in the long run [8, 9]. When coal dust penetrates the soil, it can change the physical and chemical properties of the soil and impair plant growth and development [10, 11]. Accordingly, the environmental protection department has placed a high priority on coal mine dust, which is the major cause of air pollution in mining areas. Numerous studies have been carried out on the pollution status and diffusion law of coal dust all over the world. Currently, field measurements [12], numerical simulations [13], and hyperspectral remote sensing [14] are the main research methods used to do this.

The field measurement method involves evaluation of the dust pollution during the mining process in conjunction with the air pollution index measured during coal mining and the meteorological data for the same period. However, it is not feasible to carry out field measurements in mining areas due to the limitations imposed by natural and man-made conditions [15, 16]. The numerical simulation method entails using a diffusion model to fit the relationship between the dust diffusion and meteorological factors. The diffusion movement of dust is difficult to predict and is easily affected by various factors, such as temperature, humidity, and wind. The numerical simulation method can intuitively determine the degrees of influence of the various factors on the dust diffusion. However, this method requires the collection of a large amount of monitoring data and is time-consuming [17–19]. Hyperspectral remote sensing uses hyperspectral images to monitor ground objects in the coal dust pollution area and analyzes the coal dust diffusion law using a combination of dynamic monitoring and field measurements. This method has a good monitoring effect from the point to surface scales [20, 21], but it has a low ability for spatial intuitive interpretation of coal dust pollution. The aforementioned mechanism focuses on the hazard assessment of coal dust pollution and ignores the expression of the spatial information.

In this study, the coal dust pollution in the Wucaiwan mining area in Xinjiang was taken as the research object, the Enhanced Coal Dust Index (ECDI) based on the spectral characteristics of the coal dust was constructed, and the degree of coal dust pollution was inverted using the dimidiate pixel model. The spatial and temporal distributions and causes of the coal dust pollution in the study area were analyzed, and treatment measures were suggested to provide technical support for local ecological environment protection and coal dust pollution monitoring and treatment.

## 2 Materials and methods

### 2.1 Study area

The northwestern arid area is one of the important ethnic minority areas in China. Its economic development is relatively backward. The development of mineral resources is the main economic growth mode in this area [22]. The Wucaiwan mining area is an important part of the Zhundong coalfield, which is the largest packaged coalfield in China [23]. There are approximately 390 billion tons of coal underground, accounting for approximately one-fifth of the total coal resources in Xinjiang [24]. In recent years, many enterprises have invested in the local coal industry. With the mining development, the coal dust pollution has become increasingly serious. In addition, serious soil erosion has also occurred in this area. If the environmental impact and ecological damage caused by the coal dust pollution are not studied and mediated, it will inevitably have serious consequences [25]. Therefore, the Wucaiwan open-pit mining area was selected as the study area.

**Fig 1:**
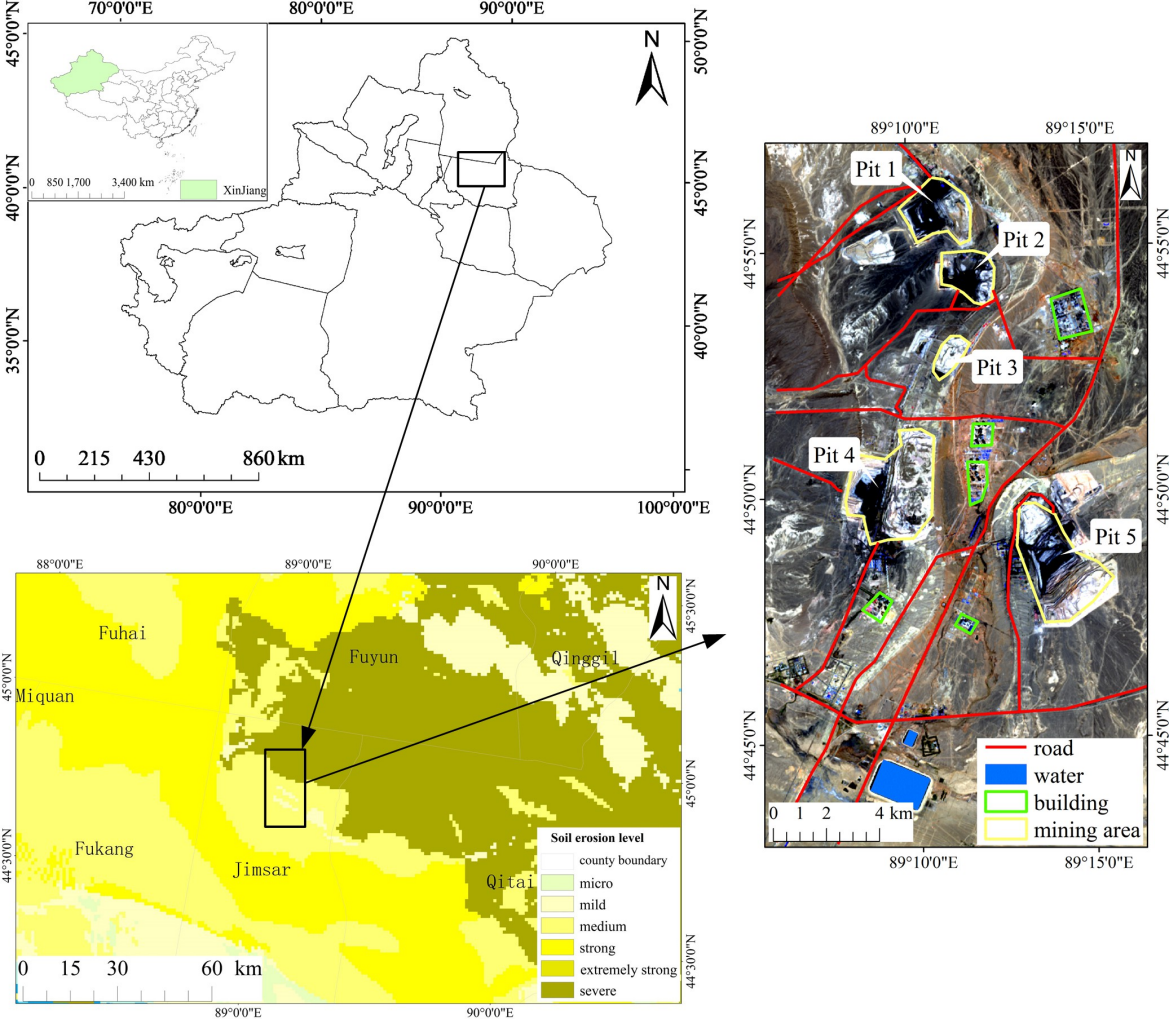
Map showing the location of the study area.

The Wucaiwan open-pit coal mine area is located in Jimusar County in the eastern part of the Junggar Basin (44°42’–44°57’ N, 89°5’–89°16’ E), Xinjiang Province, the People’s Republic of China (Fig 1) [23]. The coal bearing area is 901.05 km^2^ [26]. There are five large open-pit coal mines distributed in an L-shape in the mining area, with crisscrossing roads and convenient transportation [27]. There is no large fault structure and less seismic activity in the depression [28]. The terrain is flat and open, with an elevation of 300–600 m. The land has very sparse vegetation, with no woodlands or farmland. The area is situated in the hinterland of Eurasia, i.e., far from the ocean, and the climate is a typical extreme drought continental climate. It is hot and dry during the summer and cold in the winter. Based on the data provided by the Jimusar meteorology station, the annual average temperature is 7°C, the precipitation is 113.5 mm, the evaporation capacity is 2050 mm, and the annual average number of sunshine hours is 2950 h. Northwest and southeast winds prevail in the mining area all year round, and the maximum wind speed can exceed 40 m/s. There is no surface runoff throughout the year in this area [29, 30].

### 2.2 Data

According to a previous study [31], the Zhundong coal power and coal chemical industry park was constructed and began operation in 2010. In 2014, relevant policies on environmental governance of mining areas were announced, and the first environmental monitoring station was constructed. The Zhundong area was chosen as the national modern coal chemical industrial demonstration area in 2018, and thus, 2010, 2014, and 2018 correspond to the three important stages of the development of the mining area. The latest situation can be obtained from remote sensing images acquired in 2021, which has important practical significance for this study.

Landsat5 Thematic Mapper (TM) and Landsat8 Operational Land Imager (OLI) data are remote sensing images with a spatial resolution of 30 m and a temporal resolution of 16 days [32]. Their running time period includes the time period we analyzed in this study, and a series of Landsat satellite images have achieved global coverage. They have a great deal of band information and can be used to calculate a variety of remote sensing indexes. Therefore, in this study, a Landsat5 image taken in 2010 and Landsat8 images taken in 2014, 2018, and 2021 were selected for use in this study. The Landsat images were downloaded from the EROS Center (http://eros.usgs.gov/#).

The Gaofen-2 satellite (GF-2) is the first civil optical remote sensing satellite with spatial a resolution of better than 1 m independently developed in China. A GF-2 image with a high spatial resolution provides good verification data, which are often used to verify the accuracy of the dynamic monitoring of Landsat satellite images via visual interpretation [33]. Therefore, GF-2 images taken in 2018 were used as auxiliary reference data for the verification in this study. The GF-2 data covering the study area were acquired from the China Centre for Resources Satellite Data and Application (CRESDA). Basic information about each image is presented in Table 1.

**Table 1:** Specifications of the satellite data used in this study.

Acquisition date	Satellite	Path	Row	Cloud cover/%
2010.08.13	Landsat5-TM	142	29	0.01
2014.06.05	Landsat8-OLI	142	29	0.81
2018.09.04	Landsat8-OLI	142	29	1.34
2021.06.08	Landsat8-OLI	142	29	7.69
2018.09.11	GF-2	81	124	0.00

### 2.3 Data preprocessing

Given that the series of Landsat images was topographically and geometrically corrected before uploading, the preprocessing in this study only included radiometric calibration, atmospheric correction, and clipping of the study area. All of the subsequent analyses were conducted within the clipped images.

### 2.4 Calculating the normalized difference coal index and ECDI

In this study, the coal dust pollution in the Wucaiwan mining area was investigated. Hence, the coal dust information needed to be gathered. A previous study [34] has shown that the near-infrared band (0.845–0.885 μm, NIR) can be used to estimate biomass and identify moist soils, which can be used to distinguish minerals such as coal mines. The main function of short wave infrared band 1 (1.560–1.660 μm, SWIR1) is to identify roads, bare soil, water, and vegetation and to identify underlying buildings and structures polluted by coal dust. Shortwave infrared band 2 (2.100–2.300 μm, SWIR2) is mainly used to identify bare land and coal mines and to identify vegetation and moist soil. Therefore, an identification index for coal dust pollution can be constructed using band operations to determine the range of the coal dust pollution in the mining area.

According to the spectral profile of coal, Ma [35] selected the NIR and SWIR1 bands to establish the Normalized Difference Coal Index (NDCI), which may reflect the distribution of the coal dust pollution to a certain extent. The NDCI is calculated as follows:

$$NDCI=(Band_{SWIR1}-Band_{NIR})/\left(Band_{SWIR1}+Band_{NIR}\right).$$ (1)

The test results show that although the NDCI results for the study area can identify the coal dust-contaminated areas to a certain extent, some non-coal dust-covered areas are also identified as coal dust-contaminated areas. The reason for this is that the surrounding ground objects (rocks and soil) interfere with the sensor when receiving the reflection information about the coal, resulting in inaccurate classification results. The SWIR2 band can effectively and accurately identify rocks and minerals, and thus, a new coal dust index was constructed based on the SWIR2 band data. Referring to the method of constructing a remote sensing index used by other scholars [36], the equation for calculating the ECDI was obtained through experiments, and is as follows:

$$ECDI=\left(Band_{SWIR1}{}_{}-Band_{NIR}{}_{}+Band_{SWIR2}\right)/\left(Band_{SWIR1}+Band_{NIR}-Band_{SWIR2}\right).$$ (2)

### 2.5 Image binarization

To highlight the areas polluted by coal dust in the study area, it was necessary to binarize the ECDI image. Image binarization is the process of setting the gray values of the pixels in the image to 0 or 255 by selecting an appropriate threshold, that is, the entire image exhibits an obvious black-and-white effect. The obtained binarized image can still reflect the overall and local characteristics of the image [37].

Based on the statistical data for the ECDI image and visual interpretation results (S1–S4 Texts), 0.4 was determined to be the appropriate threshold to divide the ECDI image. The area with ECDI values of greater than 0.4 was considered to be polluted by coal dust, and the pixels became black (gray value of 0). The area with ECDI values less than 0.4 was considered to be free of pollution, and the pixels became white (gray value of 255). In this way, the amount of image data was reduced to obtain the scope of the coal dust pollution.

### 2.6 Dimidiate pixel model

As is well known, the Normalized Difference Vegetation Index (NDVI) is a vegetation index that reflects vegetation growth, while the ECDI is an index that reflects the accumulation of surface coal dust. The NDCI and ECDI are constructed in a similar way; therefore, the dimidiate pixel model of vegetation coverage can be used to estimate the degree of coal dust pollution in the mining area. If each pixel of the image is regarded as a mixed pixel of coal dust and bare soil, the following formula can be obtained [38]:

$$S=S_{C}+S_{S}\;$$ (3)

where S is the information contained in each pixel, S_C_ is the information about the coal dust, and S_S_ is the information about the other parts.

For a single pixel, the coal dust coverage of the pixel is defined as the proportion of the coal dust coverage area, which is expressed as F_CD_. Then, the proportion of the area covered by bare soil is 1- F_CD_. S_coal_ represents that the entire pixel is covered by coal dust, and S_soil_ represents that the entire pixel is covered by bare soil [40]. Then, the calculation formulas of S_C_ and S_S_ can be obtained:

$$S_{C}=F_{CD}*S_{coal}$$ (4)

$$S_{S}=\left(1-F_{CD}\right)*S_{soil}$$ (5)

Substitute Equations (4) and (5) into Equation (3), the coal dust coverage can be calculated as follows [40]:

$$F_{CD}=\left(S-S_{soil}\right)/\left(S_{coal}{}_{}-S_{soil}\right)$$ (6)

By replacing S with ECDI in Equation (6), we obtain Equation (7):

$$F_{CD}=(ECDI-ECDI_{soil})/(ECDI_{coal}-ECDI_{soil})$$ (7)

ECDI_soil_ is the ECDI value of the bare soil portion of the ECDI image, and ECDI_coal_ is the ECDI value of the coal dust pollution portion.

Based on the statistical analysis results for the ECDI (S1–S4 Texts), after many experiments, ECDI_soil_ and ECDI_coal_ values of 0.6 and 0.4, respectively, were substituted into Equation (7). The band math tool was used to calculate the dimidiate pixel model images of the ECDI.

### 2.7 Ordinary kriging

Kriging is the basis of geostatistics and is an important tool for exploring the spatial correlation between geographical phenomena. Among the various kriging methods, the ordinary kriging method is the most widely used. It is an interpolation method that uses the original known data for regionalized variables and the structural features of variance functions to make linear unbiased optimal estimates of the values in unknown regions [39].

To objectively analyze the accuracy of the ECDI in identifying coal dust, 198 points were randomly selected in the study area using the Create Random Point tool in ArcGIS 10.2 (S1 File). Ordinary kriging interpolation was performed on the ECDI values of the random points and cross-validation was carried out.

## 3 Results

### 3.1 Comparison of NDCI and ECDI

#### 3.1.1 Comparison of NDCI and ECDI images

**Fig 2:**
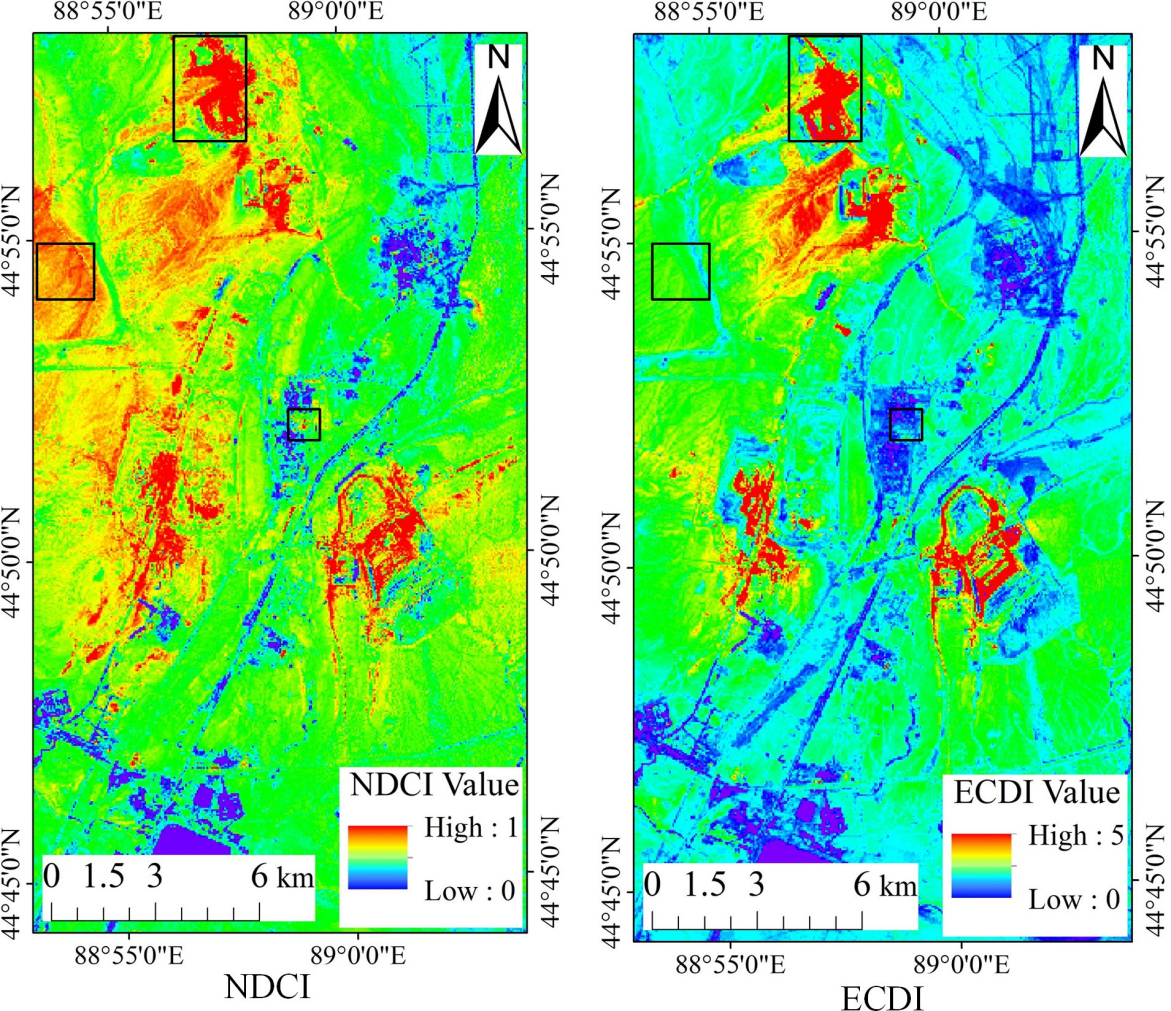
Comparison of the NDCI and ECDI images.

The NDCI and ECDI images of the study area were calculated using the above-described steps. To more clearly compare the effects of the two indexes in coal dust information extraction, two images were opened using the ENVI 5.3 software, and the rainbow ribbon in the color change table was used to represent the NDCI and ECDI images (Fig 2).

By comparing the color differences between the ECDI and NDCI images (Fig 2), it was found that in the NDCI image, red and green are widely distributed over a large area, and the other colors account for smaller areas. The area of the color gradient and transition is small. In the ECDI image, the red areas are mainly concentrated near the five pits, the green areas are relatively small, and the gradients and transitions of the various colors are more even.

#### 3.1.2 GF-2 images verify the coal dust pollution recognition effect of the ECDI

**Fig 3:**
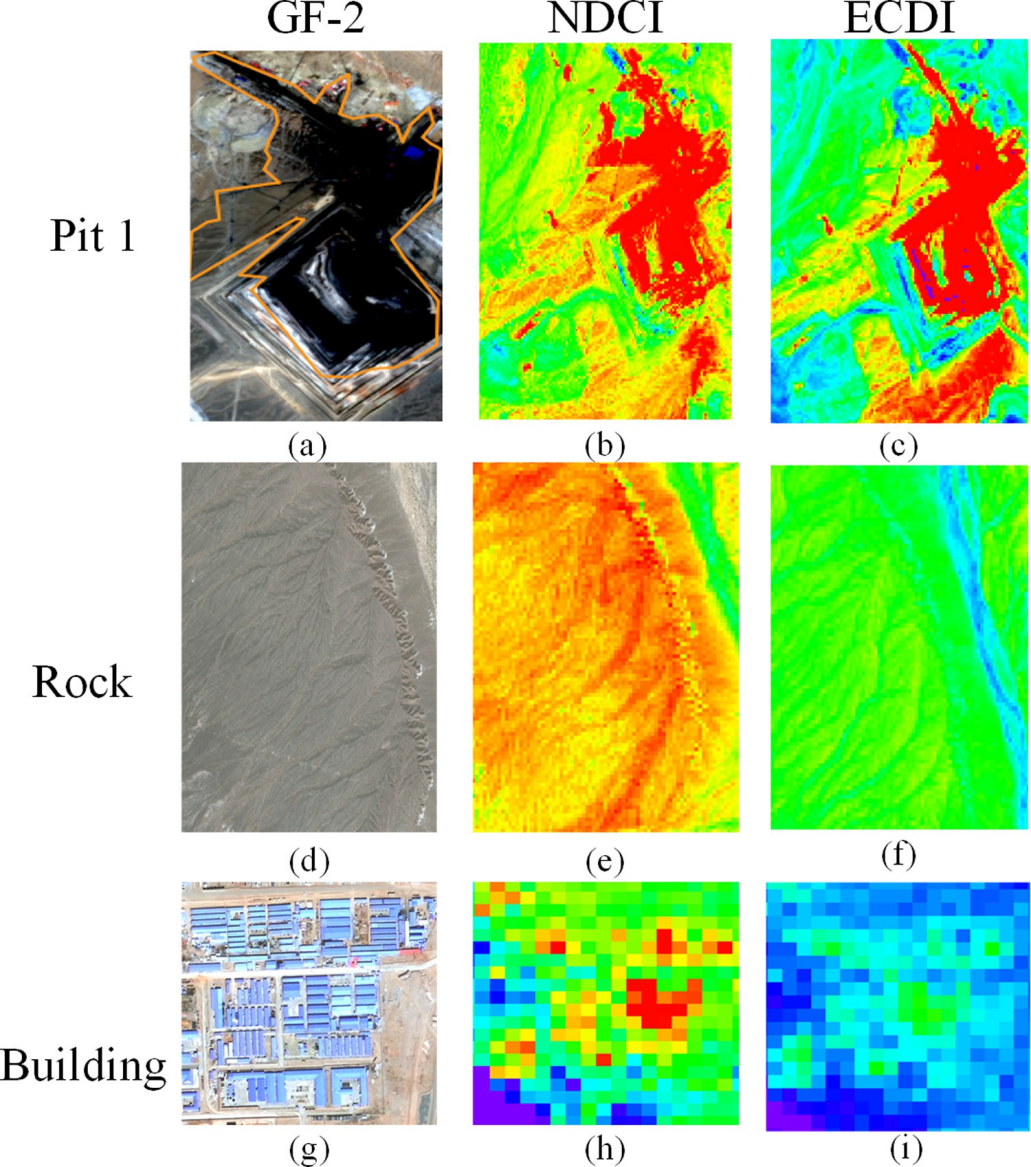
Comparison of GF-2, NDCI, and ECDI images containing coal dust, rocks, and buildings.

The recognition ability of the above image operation results in the easily confused area of the local underlying surface needs to be further verified. GF-2 images taken in 2018 were used to verify the coal dust monitoring effects of the NDCI and ECDI. Since buildings and rocks are easily confused with coal dust information, the same areas containing buildings, rocks, and Pit 1 in the three images are selected for comparison (black box in Fig 2). The comparison results are shown in Fig 3.

As can be seen from Fig 3, the coal dust pollution area around Pit 1 in the NDCI and ECDI images is shown in red, and the spatial distribution of coal dust in the ECDI image is basically consistent with the GF-2 image, indicating that the ECDI can accurately identify the scope and location of the coal dust pollution. In addition, the rocks and buildings are shown in green and blue in the ECDI data and red and yellow in the NDCI data. It can be seen that the NDCI cannot distinguish between buildings, rocks, and coal dust, while the ECDI can greatly reduce the wrong identification of rocks as coal dust.

#### 3.1.3 Comparison of spectral profiles of coal dust, rocks, and buildings

**Fig 4:**
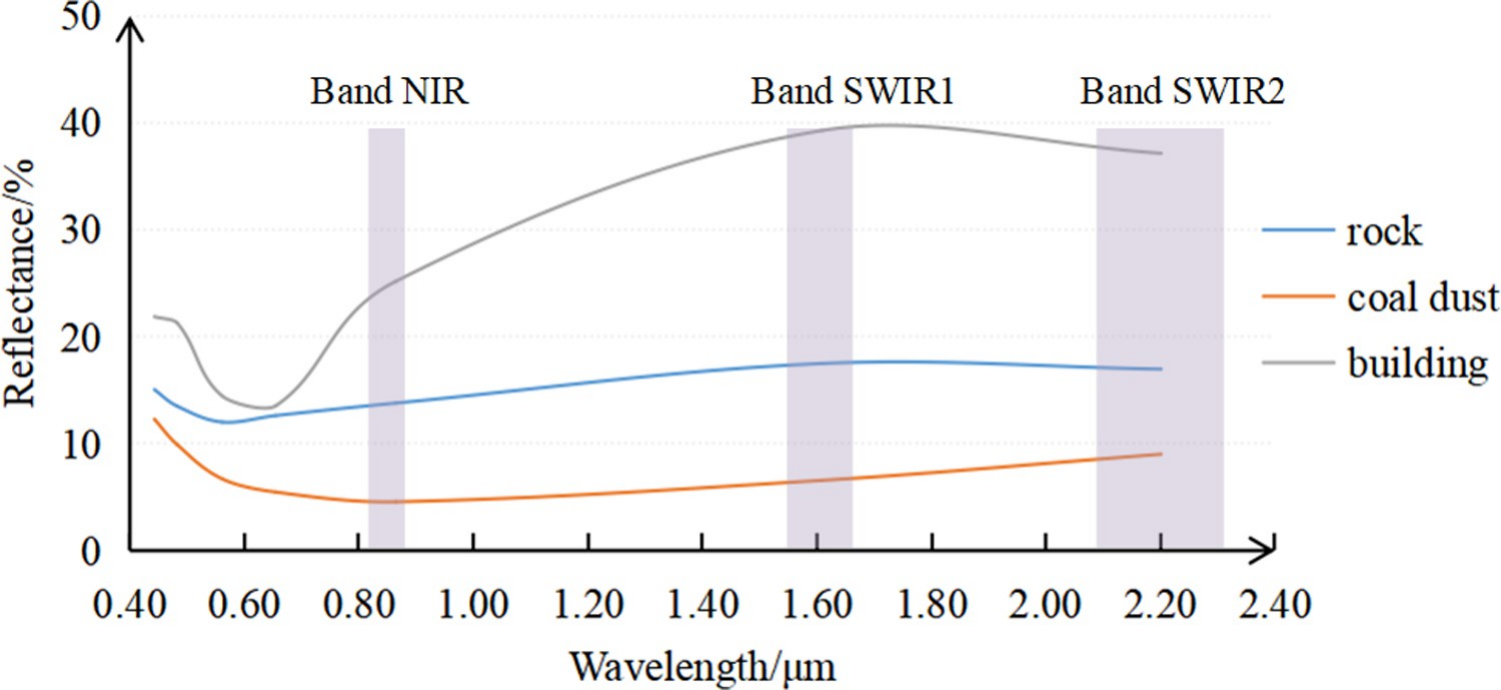
Spectral profiles of coal dust, rocks, and buildings.

To explore the reasons why the ECDI can distinguish between coal dust, rocks, and building, the ENVI5.3 software was used to draw the spectral profiles of these three types of ground objects (Fig 4).

Fig 4 shows that the spectral profiles of coal dust and rocks are relatively smooth, and their reflectance changes steadily within 0.43–2.2 μm. The spectral profile of the buildings fluctuates greatly, and the reflectance varies greatly within different bands. In the coastal (0.433–0.453 μm) and visible (0.45–0.68 μm) bands, the spectral profile of the rocks is similar to those of the buildings and coal dust, and the difference in their reflectance values is small. However, in the NIR, SWIR1, and SWIR2 bands, the reflectance values of the three types of ground objects vary greatly. The reflectance of the buildings increases to approximately 40%, while that of the rocks is close to 20%, and that of the coal dust is still less than 10%. Therefore, the ECDI created by these three bands expands the difference in the spectral information of the coal dust and surrounding ground objects, reduces the influence of foreign matter in the same spectrum, and has a stronger coal dust identification ability than the NDCI.

#### 3.1.4 Comparison of value ranges of the NDCI and ECDI

**Fig 5:**
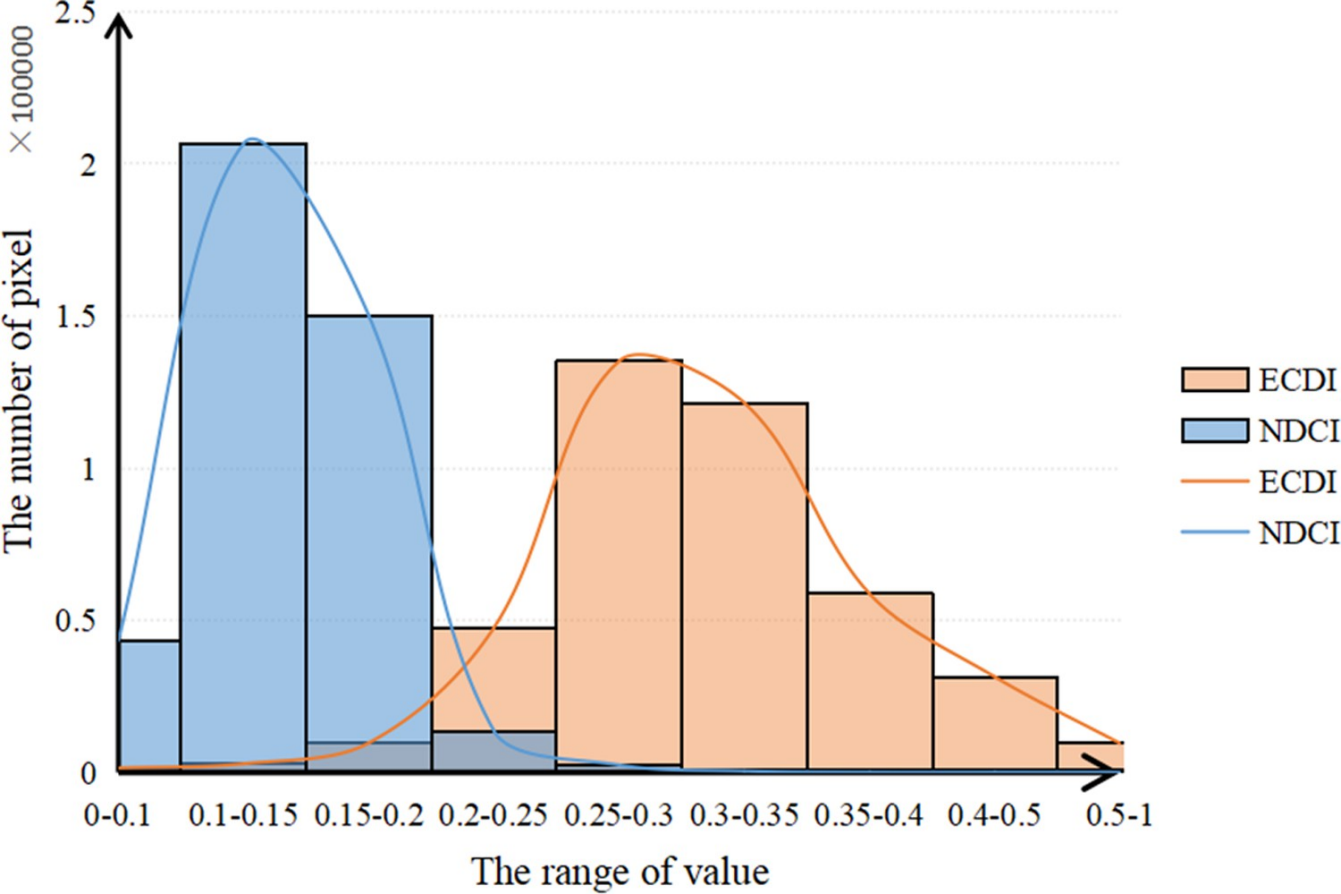
Value ranges of the NDCI and ECDI.

To further illustrate the advantages of the ECDI in coal dust information extraction, after normalizing the two indexes, the NDCI and ECDI values of each pixel in the study area were counted using the data statistical analysis tool in the ENVI 5.3 software (S3 and S5 Texts), and their pixel numbers were calculated to obtain Fig 5.

Fig 5 shows that the NDCI values were mainly 0.1–0.2, but the ECDI values were mainly 0.2–0.5. The ECDI histogram approximately obeys a normal distribution. Thus, the ECDI image is more in line with the actual situation on the ground and is suitable for remote sensing monitoring of coal dust pollution.

### 3.2 Cross-validation

**Fig 6:**
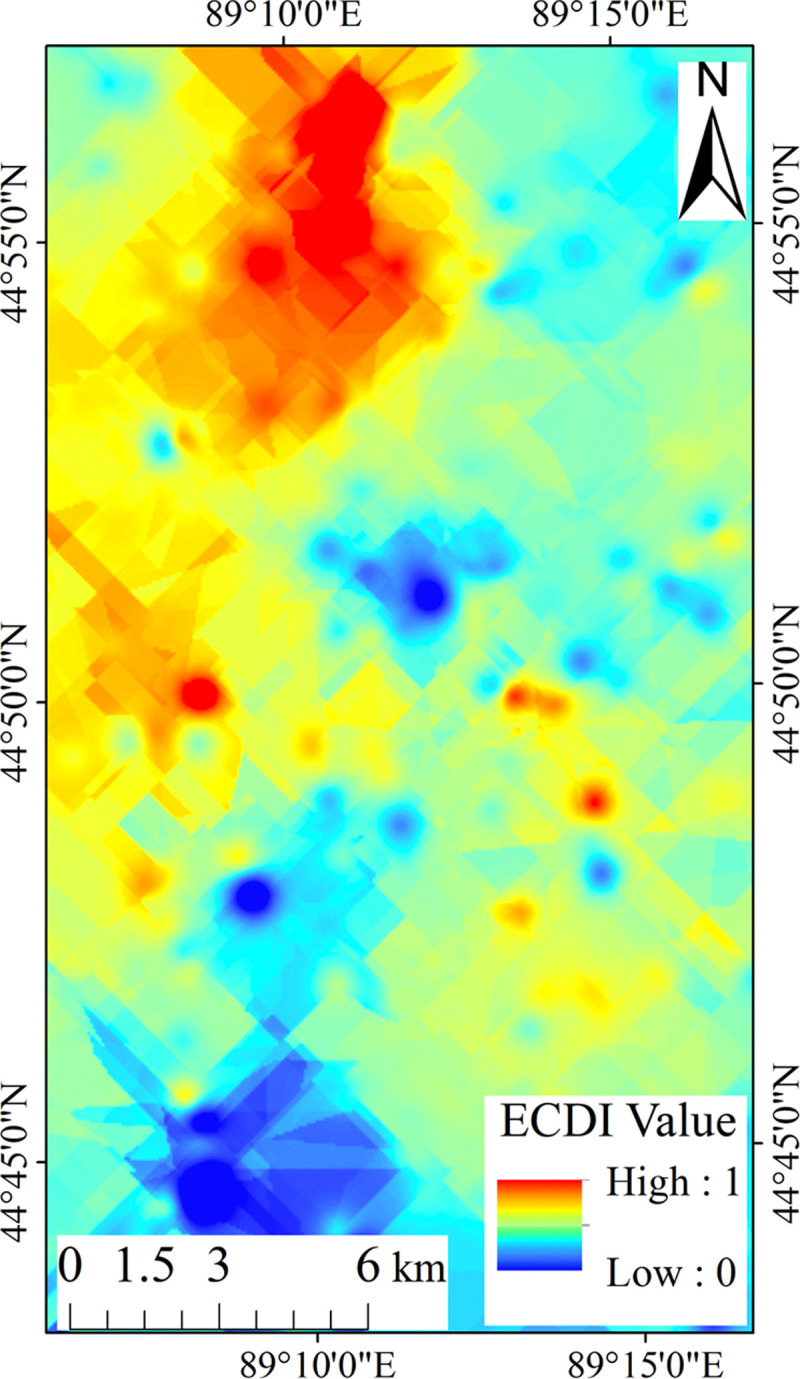
Kriging interpolation image for the ECDI.

The ordinary kriging interpolation results for the ECDI are shown in Fig 6,

As can be seen from Fig 6, the results of the ordinary kriging interpolation are basically the same as the original ECDI image, indicating that the interpolation results are reasonable and the model selection is appropriate.

The results of the cross-validation show that the Root Mean Square (RMS) of the ordinary Kriging interpolation results is 0.066, the Mean Standardized (MS) value is 0.020, the Standardized Root Mean Square (SRMS) is 0.852, and the Average Standard Error (ASE) is 0.086. The values of each index in the verification results basically meet the requirements of the optimal model, indicating that the results of the ordinary kriging interpolation are reliable, and the ECDI has a good recognition effect for coal dust.

### 3.3 Dynamic analysis of coal dust pollution in 2010–2021

**Fig 7:**
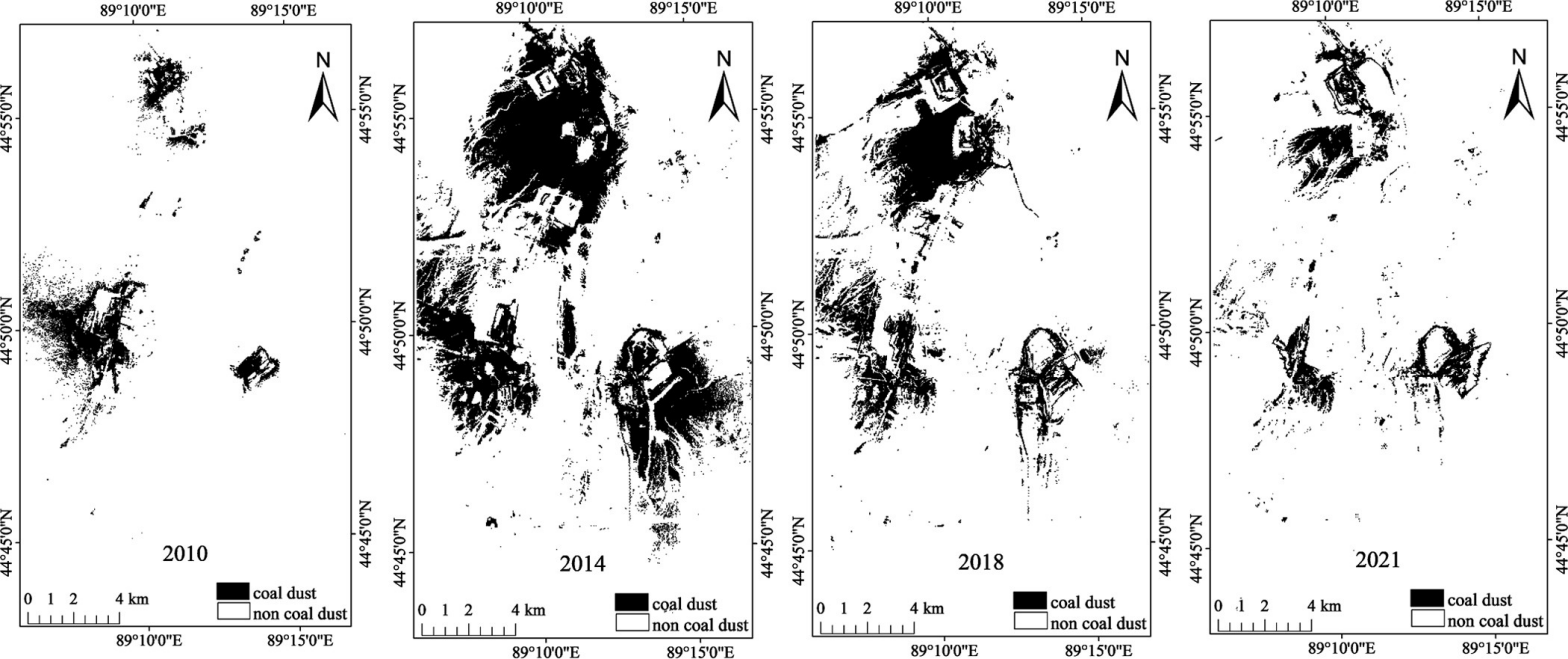
Binary images of the ECDI in 2010, 2014, 2018, and 2021.

**Fig 8:**
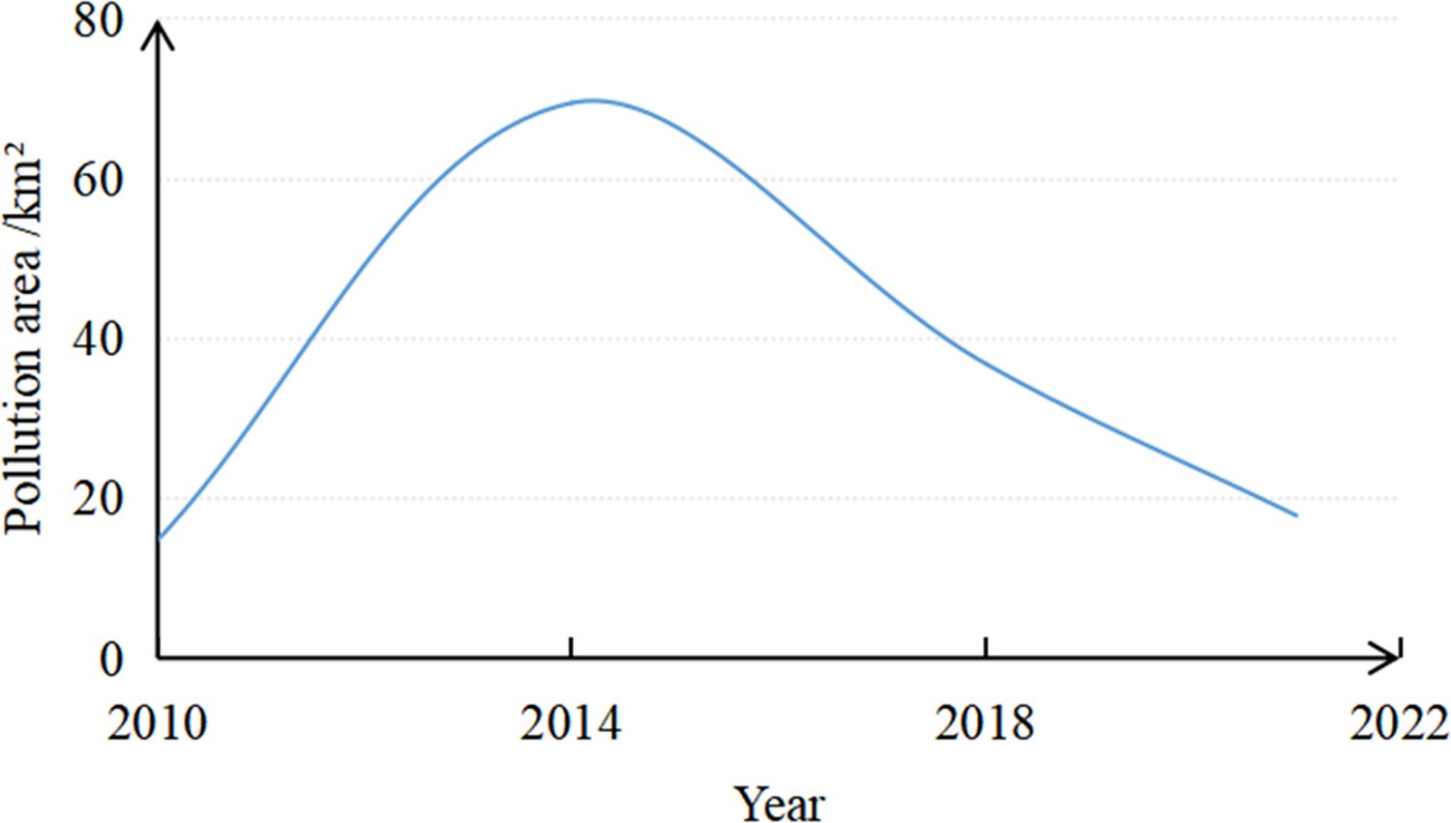
Changes in the coal dust pollution from 2010 to 2021.

The ECDI binary images of the study area acquired in 2010, 2014, 2018, and 2021 were obtained using the band math function in the ENVI 5.3 software (Fig 7). These four images were statistically analyzed, and the number of coal dust pixels counted was multiplied by the spatial resolution (30 m) to obtain the area and proportion of the coal dust pollution in the mining area in each year (Table 2). The total area of the study area was 380.95 km^2^. Then, a line chart (Fig 8) of the change in the pollution area was drawn according to the results presented in Table 2. Based on Figs 7 and 8 and Table 2, the changes in the coal dust pollution in the mining area from 2010 to 2021 were analyzed.

**Table 2:** Statistics of coal dust pollution area.

Year	2010	2014	2018	2021
**Area ratio of coal dust pollution (%)**	3.88	18.24	9.67	4.69
**Pollution area (km** ^**2**^ **)**	14.77	69.49	36.85	17.85
**Area ratio of no pollution (%)**	96.12	81.76	90.33	95.31
**No pollution area (km** ^**2**^ **)**	366.18	311.46	344.10	363.10

Fig 7 shows that the spatial and temporal variations in the coal dust pollution in the study area included the following three stages.

In the first stage (2010–2014), the coal dust pollution area around Pit 4 was the largest in 2010, and the pollution on the road next to Pit 4 was serious. In 2014, the coal dust pollution areas around Pits 1, 2, 3, and 5 significantly increased compared with those in 2010, and this pollution was basically distributed downwind of the pits. It was also observed on the road leading to the areas outside of Pits 1 and 2.

In the second stage (2014–2018), in 2018, the coal dust pollution areas around Pits 1, 2, 3, and 5 significantly decreased compared with those in 2014, and the pollution around Pit 4 remained basically unchanged. However, there was pollution on the road near Pit 1. It can be seen that some measures had been taken to control the coal dust pollution, and the environmental quality of the mining area had been improved.

In the third stage (2018–2021), compared with those in 2018, the coal dust pollution areas around the five mining pits in the study area were significantly lower in 2021, especially around Pits 1–4. Most of the coal dust pollution was concentrated in each mine, and there was almost no coal dust on the road, but Pits 1 and 2 were still more polluted than the other mines.

According to Table 2 and Fig 8, overall, the coal dust pollution area in the mining area initially increased and then decreased. Specifically, the coal dust pollution area in the study area has increased from 14.77 km^2^ in 2010 to 69.49 km^2^ in 2014. After 2014, environmental treatment began in the mining area, and remarkable results were achieved. By 2018, the pollution area had been reduced to 36.85 km^2^. By 2021, the pollution area had decreased from 36.85 km^2^ to 17.85 km^2^.

### 3.4 Distribution of coal dust pollution

**Fig 9:**
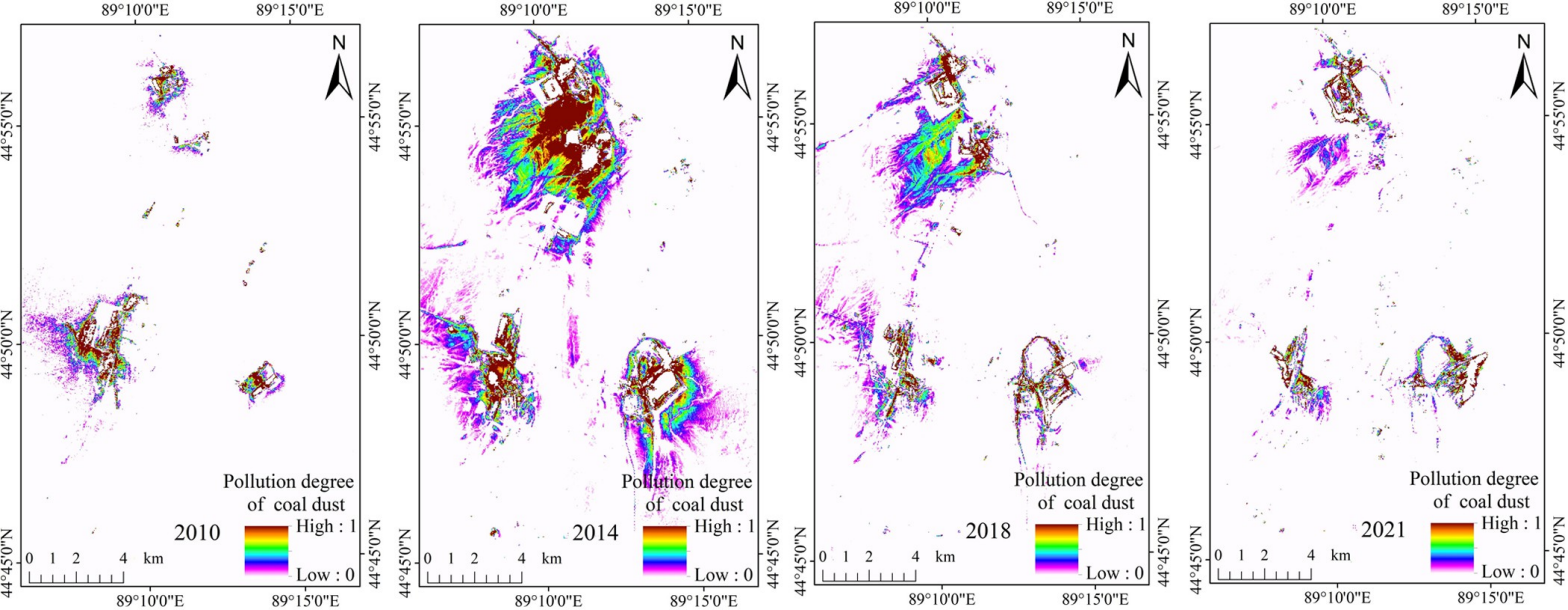
Dimidiate pixel model images of the ECDI in 2010, 2014, 2018, and 2021.

After obtaining the dimidiate pixel model image of the ECDI, the appropriate color ribbon was selected in ArcGIS10.2 software to stretch and render these images so that the color depth represented the degree of the coal dust pollution (Fig 9).

The dark color band in Fig 9 represents the most serious part of coal dust pollution, which is distributed around several pits and roads. The degree of coal dust pollution gradually decreases as the color band changes from deep to light. This shows that the closer to the pits and roads, the higher the degree of coal dust pollution.

## 4 Discussion

### 4.1 Distribution and causes of coal dust pollution

The natural causes of coal dust pollution are as follows. (1) When a coal mine is not developed, the coal is deeply buried underground, and coal dust pollution is rarely formed on the surface. Most coal dust pollution on the surface of an open-pit coal mine is produced during the mining, transportation, and processing. (2) Fig 9 demonstrates that the coal dust pollution was located to the south of the pits and around them, which may be related to the prevailing northwest wind. (3) The type of coal itself also affects the area polluted by coal dust. Different coals produce various amounts of coal dust, and the size of the coal dust particles also varies. When the coal dust particles are small, the effect of gravity is smaller, and thus, they float in the air. When the coal dust particles are large, the effect of gravity is larger, and the air cannot carry the mass of the coal dust particles. Accordingly, the coal dust will settle and gather on the underlying surface and is detected by remote sensing satellites.

The anthropogenic causes are as follows: (1) Due to the increase in the mining intensity of each pit, the frequency of coal trucks traveling to and from the mining area increases, and the coal dust diffuses during transportation, resulting in a large area of coal dust pollution around the pit and on the road. (2) Coal dust diffusion occurs during the coal mining process. For example, coal dust will diffuse during blasting and excavation in a coal mine, forming a large area and deep pollution around the pit. (3) After the coal is transported to the coal storage plant for storage, the moisture decreases, intensifying the flying and diffusion of coal dust in the air. (4) Coal dust pollution will also occur when processing the coal due to the equipment design, operation, and other problems. For example, a fast conveyor belt speed and improper operation of the equipment by the management personnel. (5) Dust removal and avoidance measures in mining areas, along transportation routes, and in coal processing plants are not enough and are not strictly managed, and the coal dust is not cleaned up in time, resulting in pollution.

### 4.2 Control measures for coal dust pollution

Recommended measures to control coal dust pollution are as follows:

1. The method of mining whilst recovering [40] should be adopted. Mining coal whilst restoring the ecological environment and treatment during coal mining should be carried out by layered stripping and staggered backfilling according to the surrounding environment and geological conditions of the open-pit coal mine.
2. Dust prevention and suppression should be carried out. Several studies [41] have shown that wetting the raw coal during storage can inhibit the diffusion of coal dust. A dust remover or water mist dust removal system should be installed in the coal storage plant, and a dust prevention and suppression wall and green plant belt should be established in the mining area to prevent coal dust pollution in the mining area, on the roads, and in the coal processing plants. Therefore, appropriate dust prevention and avoidance facilities should be established according to the local economic situation, geographical conditions, and distribution of the mining area, and effective coal dust pollution control policies should be implemented to reduce coal dust pollution.
3. Coal mine processing equipment should be improved. The sealing device should be efficiently operated, the accumulated coal dust should be removed in a timely manner, and secondary diffusion of coal dust should be prevented. Regarding the mining, transportation, and processing of the coal, management training for workers should be strengthened. Furthermore, the mining equipment should be inspected and maintained regularly to eliminate potential safety hazards.

From 2010 to 2021, the coal dust pollution area in the Wucaiwan mining area initially increased and then decreased because the local management department implemented effective coal dust pollution control methods and issued relevant policies and measures for ecological protection in the mining area. According to the statistics in the China Environmental Yearbook, since 2012, the Zhundong Development Zone has invested 6.1 billion yuan in environmental protection. Among them, the cumulative investment in planning was more than 10 million yuan, that in environmental infrastructure construction was 1.38 billion yuan, that in improving the environmental protection capacity was 5.74 million yuan, and that in ecological construction was 170 million yuan. One 21.8-million-ton solid waste landfill, one 180000-tons/year hazardous waste treatment center, one 5000-m^3^/day sewage treatment plant, and one 130000-m^3^ landfill have been built. A total of 4.7 billion yuan has been invested in the comprehensive improvement of the enterprise environment. All of the open-pit coal mining enterprises have hardened 120 km of roads and have built 46 closed silos and 40.6 km of closed coal conveying corridors. By 2021, nine production and construction coal mines in the development zone had planted trees and had created afforestation areas of more than 4.53 km^2^, effectively conserving water and soil. The use of 5G technology has made mine construction more green and intelligent, and the dust raising phenomenon of the open-pit coal mine has been effectively controlled. However, environmental protection should continue. We should continue the restoration of the ecological environment in the mining area, strengthen the construction of green mines, implement pollution prevention and control plans, and strengthen enterprise supervision to lay a solid foundation for economic development and ecological protection in this region.

## 5 Conclusions

The conclusions of our study are as follows:

1. The ECDI can identify and extract coal dust information better, reduce the interference of bare soil and other ground objects, and achieve macroscopic monitoring of coal dust pollution in the mining area to a certain extent compared with the NDCI. However, large-scale monitoring can only be carried out in the image revisit cycle due to the influence of the spatial and temporal resolutions, and the monitoring fluency and resulting description are insufficient. The ECDI_soil_ and ECDI_coal_ values need to be repeatedly revised through experiments in the process of using the dimidiate pixel model, which has a certain influence on the results. Currently, the results are only used as a reference for the spatial distribution of the degree of coal dust pollution, and the verification of its real concentration values needs to be studied further. In the future, more underlying surface factor parameters will be obtained through surface sampling to make a breakthrough in the study of the coal dust pollution diffusion mechanism.
2. The coal dust pollution in the mining area covered only 14.77 km^2^ in 2010. After the large-scale development of the coal mine, it increased to 69.49 km^2^ by 2014. After environmental treatment, the polluted area was reduced to 36.85 km^2^ in 2018, and in 2021, the pollution area was only 17.85 km^2^.
3. Compared with other areas, there was a great deal of coal dust pollution around the mines and on the roads, and the pollution was more serious closer to the mines and roads. Dust prevention measures need to be taken during coal transportation to reduce pollution. Various factors, such as wind, coal type, and the mining, processing, and transportation modes, affect the distribution of the coal dust pollution.

## Supporting information

S1 Text 2010 ECDI value. (TXT)

S2 Text 2014 ECDI value. (TXT)

S3 Text 2018 ECDI value. (TXT)

S4 Text 2021 ECDI value. (TXT)

S5 Text 2018 NDCI value. (TXT)

S1 File (RAR)

S2 File (RAR)
